# Construction of a ternary g-C_3_N_4_/TiO_2_@polyaniline nanocomposite for the enhanced photocatalytic activity under solar light

**DOI:** 10.1038/s41598-019-48516-3

**Published:** 2019-08-20

**Authors:** M. A. Alenizi, Rajeev Kumar, M. Aslam, F. A. Alseroury, M. A. Barakat

**Affiliations:** 1 Department of Physics, Faculty of Science, King Abdulaziz University, Jeddah, Saudi Arabia; 2 Department of Environmental Sciences, Faculty of Meteorology, Environment and Arid Land Agriculture, King Abdulaziz University, Jeddah, 21589 Saudi Arabia; 3 Center of Excellence in Environmental Studies, King Abdulaziz University, Jeddah, 21589 Saudi Arabia; 4 Faculty of Science, University of Jeddah, Jeddah, Saudi Arabia; 5 Central Metallurgical R & D Institute, Helwan, 11421 Cairo Egypt

**Keywords:** Environmental sciences, Nanoscience and technology

## Abstract

The combination of two or more semiconductor materials for the synthesis of new hybrid photocatalyst could be a good approach to enhance the visible light absorption, electron-hole (e^−^/h^+^) pair separation rate and photocatalytic decomposition of the organic contaminants. Herein, a facile *in situ* oxidative polymerization method has been used for the synthesis of visible light active g-C_3_N_4_/TiO_2_@polyaniline (g-C_3_N_4_/TiO_2_@PANI) nanocomposite for the decomposition of the congo red (CR) under the solar light irradiation. Prior to making the composite of TiO_2_ (P25) with g-C_3_N_4_ and polyaniline, a lamellar structure was generated onto the TiO_2_ brim by alkali hydrothermal treatment to enhance the surface area and adsorption properties. The PL and UV-visible analysis clearly showed the fast separation of the e^−^/h^+^ pair, and reduction in the bandgap energy of the g-C_3_N_4_/TiO_2_@PANI nanocomposite. The results revealed TiO_2_, PANI and g-C_3_N_4_ showed the synergestic behavior in the g-C_3_N_4_/TiO_2_@PANI nanocomposite and greatly enhanced the photocatalytic degradation of the CR. The photocatalytic decomposition of the CR was almost 100% for 20 mg/L at pH 5, 7 and 180 min. The reusability study of the spent catalyst showed the 90% degradation of CR after four consecutive cycles indicate that g-C_3_N_4_/TiO_2_@PANI nanocomposite is a stable and efficient catalyst. The high efficiency and reusability of the g-C_3_N_4_/TiO_2_@PANI nanocomposite could be attributed to the higher visible light absorption and sensitizing effect of the g-C_3_N_4_ and PANI.

## Introduction

Advanced oxidation process based on the heterogeneous photocatalysis has been proven its potential for the complete mineralization of the pollutants such as organic dyes. Numerous types of dyes have been reported in literature, which are widely used in paper, textile and leather industries, and are non-biodegradable causes potential damage to the human health due to toxicity^1^. Although organic dyes of synthetic origin are highly stable and non-degradable, heterogeneous photocatalysis can effectively degrade the dyes into the mineral products like H_2_O and CO_2_. Titanium dioxide is a well know semiconductor photocatalyst used in the UV mediated photocatalysis process due to its wide bandgap energy (~3.2 eV), activity under the ultraviolet region and fast recombination of the photo-generated electron/hole pairs (e^−^/h^+^). In the last decade, a large number of attempts have been taken to overcome the drawbacks of the pure TiO_2_, where, many tailored TiO_2_ based materials have been synthesized which showed improved optical efficiency and the visible light active photocatalytic properties^2–4^.

Numerous approach including the morphological alteration (lamellar structure, tubular, ribbon etc), chemical modification, heterojunction, introduction of metal, doping, dye sensitization, combining with other semiconductor and so forth have been widely investigated to the enhance photocatalytic properties of the semiconductor materials in visible light and suppressing the photoinduced e^−^/h^+^ pair recombination rate^1–10^. Recently, semiconductor polymers such as graphitic carbon nitride (g-C_3_N_4_), polyaniline, polypyrrole, polyacrylonitrile etc. have been combined with the metal semiconductors to enhance the photocatalytic properties. The conductive polymers have capability to act as active materials as well as conductive agent^5^. The polymeric structure has π-conjugated structure and their electronic properties can be easily adjusted by combining with the metal semiconductors. However, pure polymeric semiconductors as catalyst have some problems such as low surface area, layered stacking structure, and high photoinduced e^−^/h^+^ pair recombination rate^8–10^. So, its required to manipulate the electronic structure of semiconductor polymers to enhance photocatalytic activity.

In the past decade, polyaniline (PANI) and graphitic carbon nitride (g-C_3_N_4_) have been investigated extensively due to their stability, anti-corrosive nature, low bandgap energy, easy and low-cost synthesis. Particularly, g-C_3_N_4_ and PANI both have the high visible light absorption coefficient and shows the high mobility of the charge carrier. Nevertheless, the use of pure g-C_3_N_4_ has been retarded by the low efficiency of photocatalysis mainly due its fast change recombination and marginal visible-light absorption^10^. Ge *et al*.^11^ and Yu *et al*.^12^ synthesized the PANI–g-C_3_N_4_ composite by the *in situ* oxidative polymerization and investigated its application as an efficient catalyst for the degradation of the methylene blue under the visible light. The authors reported that PANI and g-C_3_N_4_ were strongly bonded and form the interface andshowed the up to 92.8% degradation of methylene blue. The major advantage of the g-C_3_N_4_ is its moderate bandgap energy (~2.7 eV) which can be easily photo-excited under the visible light, whereas PANI is a good electron donor and an excellent hole acceptor^13,14^. These characteristics of the g-C_3_N_4_ and PANI make them ideal materials to reduce the recombination rate of photoinduced e^−^/h^+^ pairs and enhanced the production of the active radical species for the achievement of the highest photocatalysis. Recently, the combination of the TiO_2_, g-C_3_N_4_ or PANI with other materials have been synthesized to make the binary and ternary photocatalyst. Lin and coworker^14^ synthesized the PANI/TiO_2_ composite via a hydrothermal approach for the degradation of the methyl orange and 4-chlorophenol under the UV-and visible light irradiation. There results revealed that the synergistic effect between PANI and TiO_2_ play an important role and showed the higher catalytic degradation of pollutants under both UV and visible light. Similarly, binary and ternary composites such as g-C_3_N_4_/TiO_2_^15,16^, g-C_3_N_4_/PANI^11,17,18^, g-C_3_N_4_/Ag/TiO_2_^19^, Ag/TiO_2_/Polyaniline^20^ etc. have been reported which showed higher interfacial charge transfer, thus enhanced photodecomposition of the organic pollutants. By considering the properties and advantages of the TiO_2_, g-C_3_N_4,_ and PANI, a novel nanocomposite can be designed for the photocatalytic applications. To the best of our knowledge, synthesis, characterization and photocatalytic applications of a ternary composite based on the altered TiO_2_, g-C_3_N_4_ and PANI is not reported in the literature.

Herein, a ternary g-C_3_N_4_/TiO_2_@PANI nanocomposite was established as novel photocatalytic material and applied for degradation of toxic dyes congo red in waster. Initially, Degussa TiO_2_ (P25) powder was altered with the 10 M NaOH to generate the defect in TiO_2_ lattice and lamellar structure of mixed phase titania and sodium titanate. We assumed that mixed phase titania and sodium titanate lamellar structure could have the better surface area for the binding with the g-C_3_N_4_ and PANI which will facilitate the higher interfacial charge separation and enhanced adsorption-photocatalytic properties for the removal of the CR molecules. The photocatalytic activity of the g-C_3_N_4_/TiO_2_@PANI nanocomposite for the degradation of the CR has been investigated under direct sunlight irradiation.

## Results and Discussion

### Characterization

**Figure 1:**
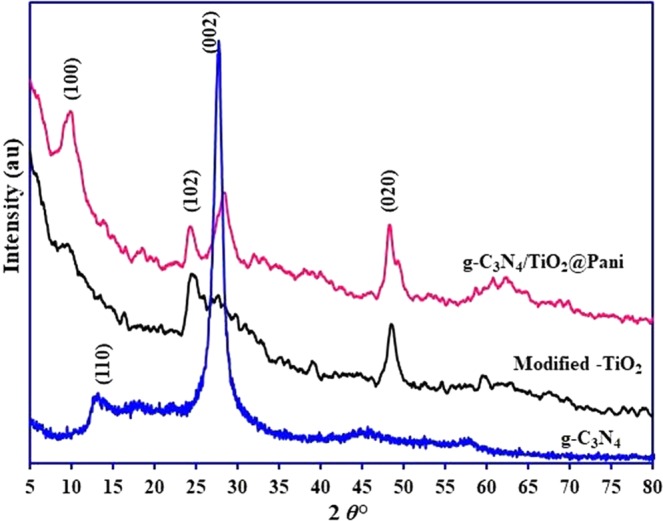
XRD pattern of modified TiO_2_, g-C_3_N_4_ and g-C_3_N_4_/TiO_2_@PANI nanocomposite.

The modification in TiO_2_ lattice structure after NaOH treatment and nanocomposite structure were analyzed by XRD, SEM, PL, and UV-visible DRS techniques. The XRD pattern of the modified TiO_2_, g-C_3_N_4_ and g-C_3_N_4_/TiO_2_@PANI nanocomposite are shown in Fig. 1. The XRD peaks for the TiO_2_ appears at 2*θ*° ≈ 27.44 (110), 36.03(101), 39.16(200), 41.23(111), 44.03(210), 54.32(211), 56.63(200), 62.76(002), 64.02(310), 69.0(301), and 69.80 (112)^7^. After the treatment with NaOH, the crystal phase of the TiO_2_ changes and new broad peaks appeared at 2*θ*° ≈ 10, 28.19, and 48.14, which are corresponding (110), (102) and (020) for the mixture of TiO_2_ and crystalline sodium titanate^21^. The XRD pattern of the modified TiO_2_ is very close to the sodium titanate nanotubes as reported by the various researchers^6,22,23^. Herein, TiO_2_ particles was not changed into the nanotubes due to designed experimental conditions such as temperature and reaction time^24,25^. The XRD pattern of g-C_3_N_4_ shows the two strong peaks centered at 2*θ*° ≈ 12.72 (110) and 27.3 (002). The XRD pattern of the g-C_3_N_4_/TiO_2_@PANI nanocomposite showed all the characteristic peaks for the g-C_3_N_4_ modified TiO_2_ with the light change in the peak position and intensity. However, no peak was observed for the polyamine probably due to its amorphous nature and a low amount in the nanocomposite. Moreover, a characteristic peak for g-C_3_N_4_ appeared at 27.3° shifted to 28.4° showed better indication with the polyamine and TiO_2_.

**Figure 2:**
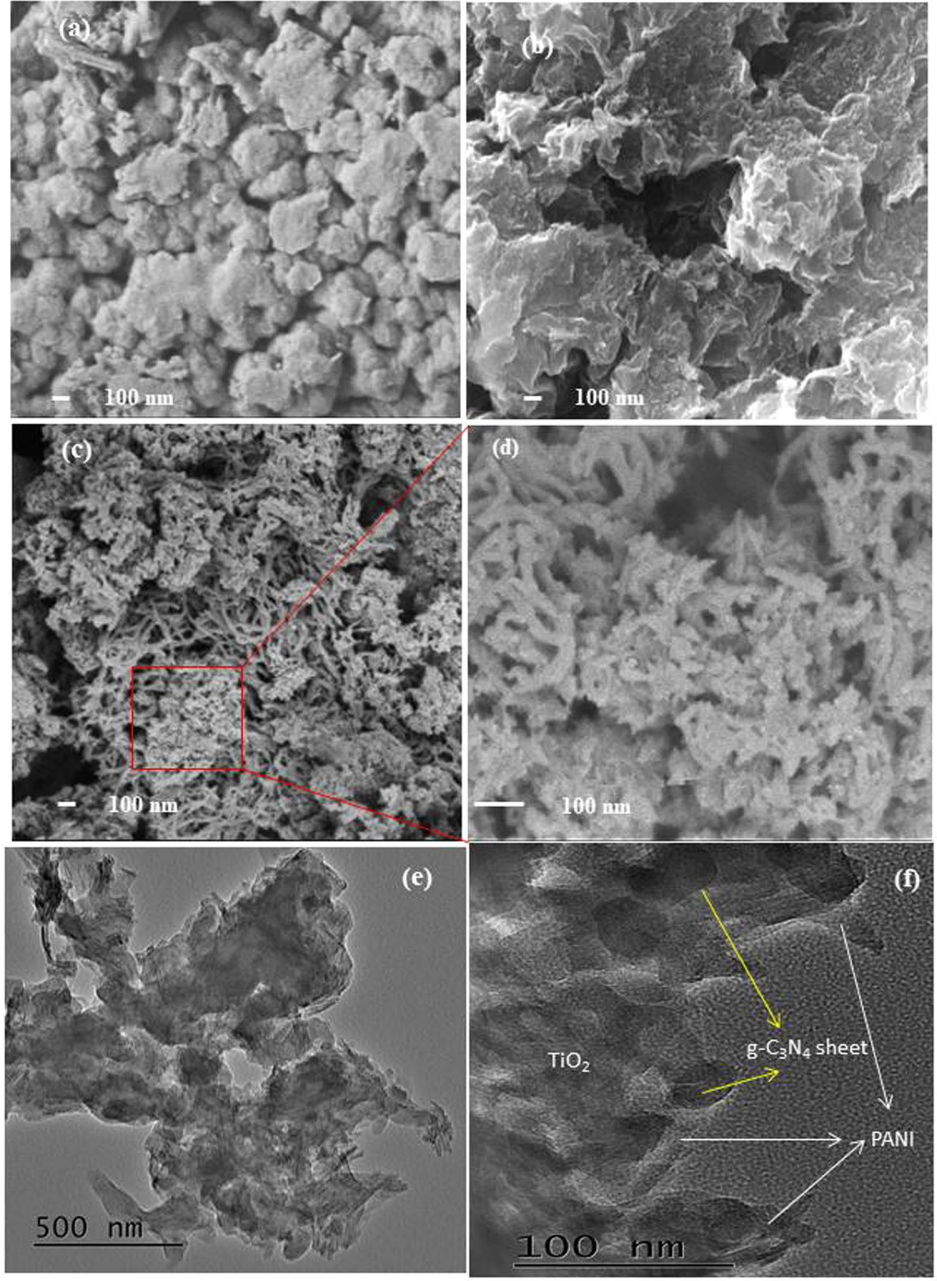
SEM images of (**a**) g-C_3_N_4_ (**b**) modified TiO_2_, and (**c**,**d**) g-C_3_N_4_/TiO_2_@PANI nanocomposite and (**e**,**f**) TEM images of g-C_3_N_4_/TiO_2_@PANI nanocomposite.

The morphology changes after each modification of the prepared materials are shown in Fig. 2. The SEM image for the g-C_3_N_4_ showing the irregular shaped structure (Fig. 2a). The SEM image for modified TiO_2_ shows the lamellar structure which was originated after the treatment of the TiO_2_ particles (Fig. 2b). The morphology of the g-C_3_N_4_/TiO_2_@PANI nanocomposite showing the lamellar TiO_2_ structure with polyaniline deposited on the surface (Fig. 2c,d). Furthermore, the distribution of the g-C_3_N_4_ and PANI on the surface of the lamellar TiO_2_ surface was analyzed by TEM. As shown in Fig. 2e,f, a lamellar structure of TiO_2_ which is well covered by the polymeric PANI and well distributed g-C_3_N_4_ sheets in the g-C_3_N_4_/TiO_2_@PANI nanocomposite.

**Figure 3:**
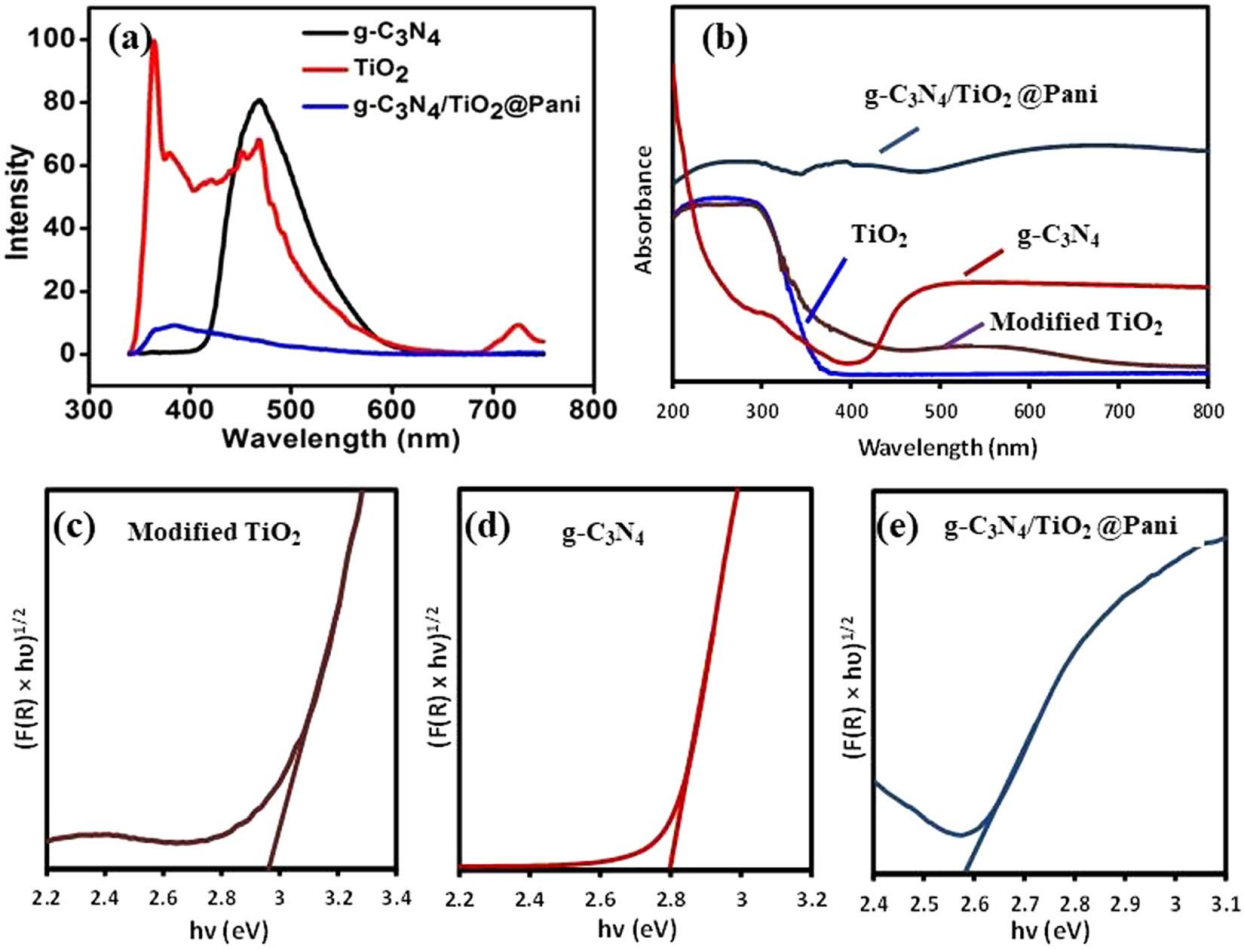
(**a**) PL spectra (**b**) UV-Visible absorbance spectrum and (**c**–**e**) (F(R) x hυ)^1/2^ versus hυ plot for bandgap energy calculation of modified TiO_2_, g-C_3_N_4_ and g-C_3_N_4_/TiO_2_@PANI nanocomposite.

Photoluminescence (PL) spectroscopy can give an idea about the recombination rate of the photo-induced electron (e^−^) and hole (h^+^) pairs which can be used to predict the photocatalytic efficiency of materials. The PL emission spectra of g-C_3_N_4_, modified TiO_2_ and g-C_3_N_4/_TiO_2_@PANI nanocomposite is shown in Fig. 3a. In the case of g-C_3_N_4_, the PL spectra shows a main peak in the ~450 nm region corresponding to the n–π* electronic transitions^26^. The highest PL intensity of g-C_3_N_4_ also indicates its highest charge recombination rate and hence poor photocatalytic efficiency. The titanate showed a few small peaks at a higher wavelength and one major peak at 362 nm region which is in the similar range to the observations of Qumar *et al*.^23^. The peaks in the range, ~475–550 nm, can be assigned to the free electron recombination process from the conduction band to the ground state recombination center^20^. PANI has been reported to cause oxygen vacancies, local distortions, strains in the lattice structure which in turns affects the optical properties^24^. The lowest PL intensity of C_3_N_4/_TiO_2_@PANI nanocomposite suggests the lowest recombination rate and thus the high lifetime of charge carriers which might consequently result in its highest photocatalytic efficiency. This also confirms that PANI apart from acting as a good light harvester, might also disperse charge carries similarly as done by conductive materials like graphene, carbon nanotubes etc.

The solid-state absorption spectra of synthesized photocatalysts were explored in the wavelength range of 200 nm to 800 nm. The diffuse reflectance absorption spectra of TiO_2_, Modified TiO_2_, g-C_3_N_4_, and g-C_3_N_4_/TiO_2_@PANI is presented in Fig. 3b. For the pure TiO_2_ in the visible range of 400–800 nm, no significant absorption peak was noticed. Compared to the pure TiO_2_, the modified TiO_2_ showed a shrill increase in the magnitude of absorption while g-C_3_N_4_/TiO_2_@PANI nanocomposite indicated the stronger and broad absorption band from ultraviolet to visible light. The absorption edge of pure g-C_3_N_4_ was around 450 nm with extensive photoabsorption which is attributed to the excitation of an electron from 2p orbital of N (valence band) to the 2p orbital of C (conduction band). The bandgap energies were also calculated through reflectance data (%R) of each synthesized photocatalysts by primarily applying the Kubelka-Munk function (F(R)) to %R data and finally plotting the (F(R) x hυ)^1/2^ versus hυ (photon energy). The Fig. 3c,d, suggests the detonated graphical assessment of the band gaps wherein the bandgap of the synthesized photocatalysts can be comprehended via the extrapolation of the onset location of the curve to the x-axis. From the Fig. 3c–e, the assessed bandgap energies for the modified TiO_2_, g-C_3_N_4_, and g-C_3_N_4_/TiO_2_@PANI nanocomposite were 2.97, 2.8, and 2.58 eV, respectively. The redshift of the absorption edge for the ternary g-C_3_N_4_/TiO_2_@PANI nanocomposite towards the higher wavelength indicating the lowering in the bandgap and enhancing the visible light absorption ability. The results showed that under the visible-light illumination the synthesized ternary nanocomposite may facilitate for the more electron transfer and can be applied to enhance the photocatalytic activities.

### Photocatalysis

**Figure 4:**
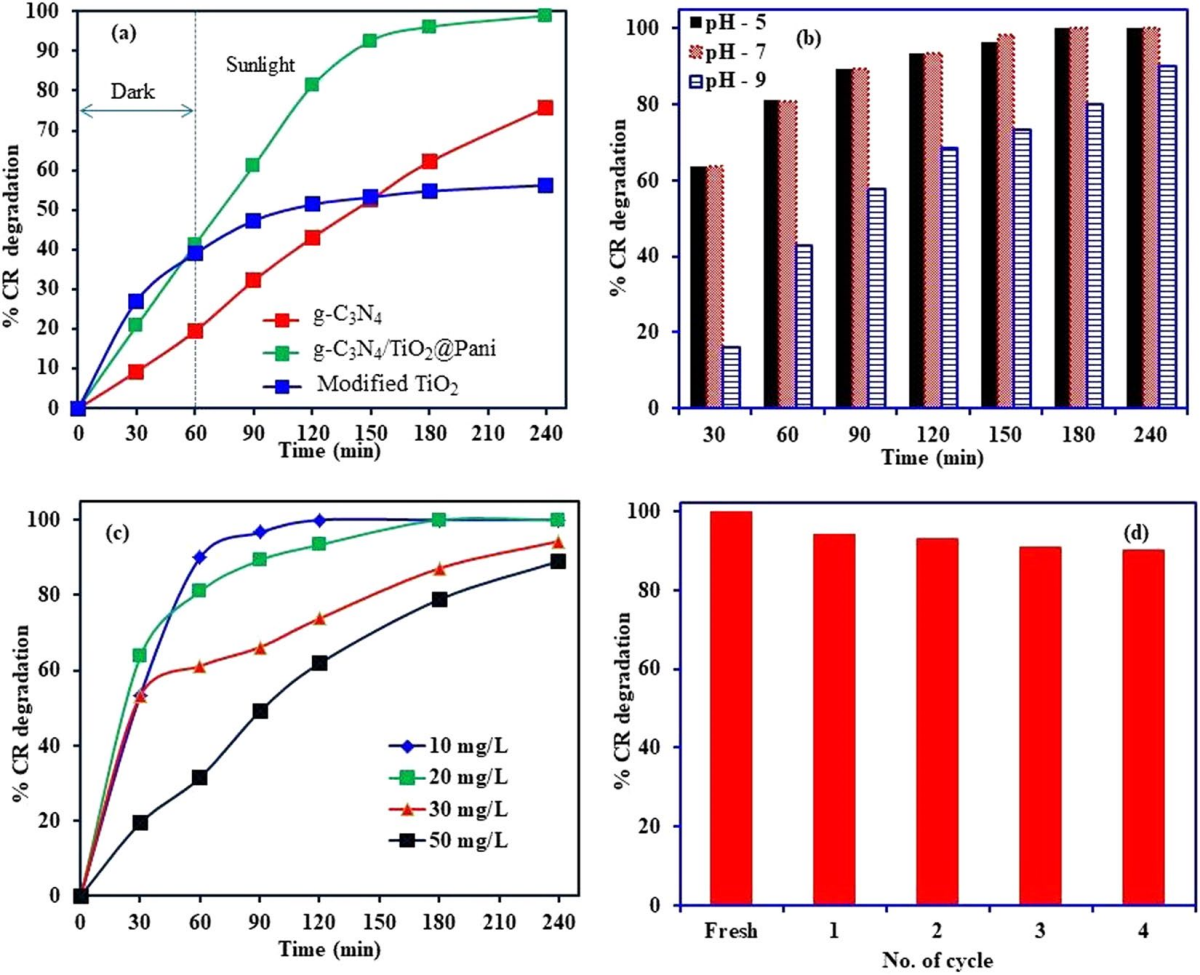
Photocatalytic degradation of CR (**a**) comparative degradation study (**b**) effect of solution pH on CR degradation onto g-C_3_N_4_/TiO_2_@PANI nanocomposite (**c**) effect of initial CR concentration on photocatalysis by g-C_3_N_4_/TiO_2_@PANI nanocomposite and (**d**) reusability study g-C_3_N_4_/TiO_2_@PANI nanocomposite.

The photocatalytic properties of the prepared materials g-C_3_N_4_, modified TiO_2_ and g-C_3_N_4_/TiO_2_@PANI nanocomposite are shown in Fig. 4a. The results showed the g-C_3_N_4_/TiO_2_@PANI nanocomposite attained the better decomposition of CR molecules compared to the g-C_3_N_4_ and TiO_2_ due to better separation of e^-^/h^+^ pair and lower bandgap energy. These results clearly show that g-C_3_N_4_/TiO_2_@PANI nanocomposite is efficient material for the degradation of CR molecules. These results are in compliance with the PL, bandgap analysis and UV-visible analysis of the g-C_3_N_4_, modified TiO_2_ and g-C_3_N_4_/TiO_2_@PANI nanocomposite. Moreover, the binding of the CR molecules onto g-C_3_N_4_/TiO_2_@PANI nanocomposite is much higher than the g-C_3_N_4_ and TiO_2_ due to its hybrid nature which contains a large number of the functional groups belongs to the g-C_3_N_4_, TiO_2_, and polyaniline. In the first step, the CR molecules present in solution quickly interact with the active site present on the g-C_3_N_4_/TiO_2_@PANI nanocomposite. While in the second step, adsorbed CR molecules degraded by the photocatalysis. Modified TiO_2_ shows a good adsorption capacity in the dark while under the sunlight irradiation a slight increase in the degradation was observed because of the poor visible light absorption properties. However, g-C_3_N_4_ shows the comparative lover adsorption of the CR molecules but under the solar light irradiation, it shows the gradual increase in the decomposition. Therefore, g-C_3_N_4_/TiO_2_@PANI nanocomposite has been selected for the further photocatalytic experiments. In order to optimize the optimum conditions for the degradation of the CR molecules onto g-C_3_N_4_/TiO_2_@PANI nanocomposite, the effect of initial CR concentration, solution pH and reaction times has been studied. The effect of solution pH on CR molecules degradation by g-C_3_N_4_/TiO_2_@PANI nanocomposite is performed at the solution pH 5, 7 and 9. The decomposition of Cr molecules onto the g-C_3_N_4_/TiO_2_@PANI nanocomposite is shown in Fig. 4b and the results demonstrated that acidic and neural medium is the most favorable conditions for the decomposition of the CR molecules. This degradation behavior of the CR molecules onto g-C_3_N_4_/TiO_2_@PANI nanocomposite can be elucidated on the basis of the surface charge and interaction between the g-C_3_N_4_/TiO_2_@PANI nanocomposite and CR molecules. The photocatalyst, g-C_3_N_4_/TiO_2_@PANI nanocomposite was synthesized under the acidic condition. Therefore, protonated polyaniline (emeraldine salt) is present in g-C_3_N_4_/TiO_2_@PANI nanocomposite which has the net positive charge on the catalyst surface which interacts electrostatically with the negatively charged CR molecules. Therefore, a higher rate of photodegradation of CR molecules is observed at the pH 5 and 7^25,27^. Additionally, the basic medium provides the more hydroxyl ions in the solution which neutralize the surface charge present on the g-C_3_N_4_/TiO_2_@PANI surface and produce the negatively charged g-C_3_N_4_/TiO_2_@PANI surface which repel the anionic CR molecules. Moreover, the basic condition decreases the oxidation potential of the hydroxyl radicals produced during the photocatalysis reaction^28^.

The aqueous solution of the dyes is the highly coloured and optical density of the solution decreases with the increase in the initial dye concentration. For the efficient photocatalytic degradation of the CR molecules, the photons and g-C_3_N_4_/TiO_2_@PANI nanocomposite must interact properly to generate the active radical species. Therefore, the effect of the initial CR concentration has been investigated to identify the optimum CR concentration at which maximum photocatalytic degradation occurs. The results depicted in Fig. 4c indicate that the decomposition of the CR molecules reduces from 100 to 89% as the initial solution concentration increases from 10 to 50 mg/L. However, the photocatalysis time is the most important fact here because almost 100% degradation is observed for 10 mg/L with 120 min while only 61.8% CR molecules are degraded within the same time. These results illustrated that g-C_3_N_4_/TiO_2_@PANI nanocomposite showed the fast decomposition of CR molecules at lower concentration and the decomposition rate decreased as the initial CR concentration increases. The may be related to the optical density of the CR solution, amount the adsorbed dye molecules and lack of the interaction between the photons and g-C_3_N_4_/TiO_2_@PANI nanocomposite^29^. As the CR concentration rises, the optical density of the solution decrees and penetration of the visible light in the solution reduces. Therefore, less number of the photons reaches to the g-C_3_N_4_/TiO_2_@PANI surface which suppresses the production of photogenerated e^−^/h^+^ pairs and active radical species. Thus, a lower degradation of the CR molecules is observed at the higher initial concentration^28^.

### Reusability

The prospective applicability of the synthesized photocatalyst for the large-scale applications can be tested on the basis of the stability and reusability. The reusability of the g-C_3_N_4_/TiO_2_@PANI nanocomposite was repeated for the CR degradation up to four cycles by taking the 0.05 g catalyst in 200 mL dye solution of 20 mg/L concentration at pH 5 and the results are shown in Fig. 4d. For the recyclability, g-C_3_N_4_/TiO_2_@PANI nanocomposite was washed every time with de-ionized water and dried at 60 °C for 12 h. The revealed that the fresh g-C_3_N_4_/TiO_2_@PANI nanocomposite showed the almost 100% decomposition of CR molecules. While the percentage decomposition of the CR was reduced up to 90.1% after the fourth cycle. This reduction in the photocatalytic efficiency of the g-C_3_N_4_/TiO_2_@PANI nanocomposite can be ascribed to the adsorbed molecules of CR or degradation product in the internal structure of the catalyst^27^. These results indicated that g-C_3_N_4_/TiO_2_@PANI nanocomposite is a stable catalyst under the applied experimental conditions.

### Comparative photocatalytic degradation of CR

The photocatalytic degradation efficiency of the various catalysts for CR molecules and other organic pollutant has been summarized in Table 1. The reported literature revealed that the degradation of the CR molecules has been performed by various types of catalysts under the different type of irradiation sources. Table 1 revealed that photocatalytic efficiency of the g-C_3_N_4_/TiO_2_@PANI nanocomposite is comparatively better than the previously reported works. Also, g-C_3_N_4_/TiO_2_@PANI also showed higher photocatalytic removal of dye as compared to the previously reported TiO_2_-PANI and g-C_3_N_4_-PANI composites. For instance, (Yazhini *et al*., 2015) used TiO_2_-PANI for degradation of MB dye and achieved maximum 69.9% of removal, Similarly, Ge *et al*., (2012) achieved up to 92% of photocatalytic removal of MB dye using g-C_3_N_4_-PANI composite. Here we achieved 100% dye removal g-C_3_N_4_/TiO_2_@PANI, thus, highest possible efficiency was achieved. Moreover, the major advantage of this work is that we used the solar light which is cost effective compared to the synthetic light.

**Table 1:** Comparative photocatalytic efficacy of various catalysts used for the removal of CR dye and other organic pollutant decomposition.

Catalyst	Pollutant	Degradation (%)	Experimental conditions					Reference
			pH	Conc. (mg/L)	Volume (mL)	Time (h)	Dose (mg)	
Ba/Alg/CMC/TiO_2_	CR	91.5	6.05	40,8	50	4	54	23
CZ-400	CR	65	7	200	20	2	40	28
Hombikat UV-100 TiO_2_	CR	97	7	70	30	0.41	30	29
WO_3_–TiO_2_/activated carbon	CR	83	7	80	50	2	400	34
TiO_2_-SWNT-P-21	CR	82	7	250	50	1.33	25	35
alginate/Fe_2_O_3_/CdS composite	CR	91.6	5.6	20	50	5	25	36
UV-TiO_2_-O_2_	CR	86	7	55	50	8	50	37
TiO_2_-PANI	DB1	69.2	7	—	—	0.5	100	38
g-C_3_N_4_-PANI	MB	92	—	10	50	2	100	39
TiO_2_/g-C_3_N_4_/G	NB	97	—		50	4	100	40
g-C_3_N_4_-TiO_2_@PANI	CR	100	7	20	150	3	30	This work

### Photocatalysis mechanism

The possible mechanism of the improved photocatalytic performance of on g-C_3_N_4_/TiO_2_@PANI nano-hybrid catalyst linked to the efficient absorbance of visible light and separation of electron hole pairs. Upon irradiation of visible light the electron/pairs get separated for both in both materials TiO_2_ and g-C_3_N_4_. The electrons in the conduction band of TiO_2_ recombine with the holes of the g-C_3_N_4_ valence band. This phenomena prevents the recombination of electrons and holes^30^. Moreover, the generated holes also transmitted to the PANI surface^31^. As a result, the electrons remained in the g-C_3_N_4_ conduction band which react with oxygen and form reactive superoxide radicals (^•^O_2_^−^) having capability to directly oxidize the dyes molecule^32^. Moreover, some of the O_2_ react with H_2_O and generate ^•^OH radicals^33^. The holes present in the valence band of TiO_2_ and surface of PANI also reacts with H_2_O producing more ^•^OH radicals. Overall, these ^•^OH radicals and electrons remained in the g-C_3_N_4_ conduction band which react with oxygen and form reactive superoxide radicals (^•^O_2_^−^) are powerful oxidative agent which efficiently degrade dye molecules as compared to that with single material i.e. TiO_2_ and g-C_3_N_4_.

## Conclusion

A ternary nanocomposite based on modified TiO_2_, g-C_3_N_4_ and polyaniline (g-C_3_N_4_/TiO_2_@PANI) was successfully synthesized via a facile *in-situ* oxidative polymerization technique. The synthesized g-C_3_N_4_/TiO_2_@PANI nanocomposite was tested as an active photocatalyst for the degradation of the congo red (CR) under the natural sunlight irradiation. The result revealed that g-C_3_N_4_ and PANI are not only suitable to alter the bandgap energy but also suppress the e^−^/h^+^ pairs recombination rate which facilitate the higher decomposition of CR molecules by g-C_3_N_4_/TiO_2_@PANI under the sunlight irradiation. The synthesized nanocomposite showed almost 100% photocatalytic decomposition of the CR molecules at 10 and 20 mg/L concentration within 120 and 180 min, respectively. As the concentration of the CR in solution increases up to 50 mg/L, the rate of photocatalysis reduces due to the poor optical density of the solution and lesser production of active radicals responsible for decomposition of CR. Moreover, the reusability study also shows that g-C_3_N_4_/TiO_2_@PANI is a stable catalyst and can be used several times without losing its efficiency. The obtained results demonstrated that g-C_3_N_4_/TiO_2_@PANI is a highly active and efficient nanocomposite which can be used for the decontamination of the organic pollutants from the wastewater.

## Materials and Method

### Materials

The congo red dye (C_32_H_22_N_6_Na_2_O_6_S_2_) and TiO_2_ (P25 phase) were obtained from fisher chemicals and Alfa-Aesar. Aniline, melamine, and oxidant potassium persulfate were obtained from Techno Pharmachem Haryana, India, BDH Ltd, and SD Fine chemical Ltd, respectively.

### Preparation of g-C_3_N_4_ and modification of TiO_2_

A yellow g-C_3_N_4_ powder was prepared by heating of melamine at 550 °C for 3 h in a muffle furnace at the heating rate of 5 °C/min as reported in our previous work^8^.

The modification of the TiO_2_ was performed according to the previously reported method^6^. Initially, 3.0 g TiO_2_ was mixed with 10 M NaOH solution and stirred for 5 h. Thereafter, TiO_2_ slurry was transferred to the hydrothermal reactor and heated for 18 h at 120 °C. The obtained white precipitate was filtered, washed with deionized water and acetone to remove the excess of the Na^+^. The white precipitate was dried at 115 °C for 18 h.

### Synthesis of g-C_3_N_4_/TiO_2_@polyanilene nanocomposite

A facile *in-situ* chemical oxidative polymerization method was followed to prepare the g-C_3_N_4_/TiO_2_@PANI nanocomposite. About 0.04 g of g-C_3_N_4_ added to 1.0 M HCl solution and stirred for 16 h. Thereafter, 1.5 g modified TiO_2_ was added and further stirred for 3 h. Then, 1 mL aniline was added to 1.0 M HCl solution and stirred in an ice bath. TiO_2_ solution was added to the aniline solution and stirred for 15 min. For the polymerization, oxidant ammonium persulfate (0.1 M, 50 mL) was added dropwise under continuous stirring. A bluish precipitate was formed and stirred for the 16 h. Thereafter, the solution was filtered and washed thoroughly with water, acetone and dried at 105 °C. A total yield of 1.79 g (90.1%) composite was obtained. A similar method was used for the synthesis of PANI in the absence of g-C_3_N_4_ and TiO_2_ and 0.39 g of the PANI was obtained. A loss of around 10% in total yield of g-C_3_N_4_/TiO_2_@PANI nanocomposite was observed. The reduction in the % yield may be due to the loss of some amount during washing of the material.

### Photocatalytic experiments

The photocatalytic decomposition of the CR molecules was performed in the batch process. Initially, a fixed amount of the prepared catalyst was added into 150 mL dye solution of known pH and concentration. The adsorption of the CR onto the prepared photocatalyst was performed in dark for 1 h to attain the adsorption-desorption equilibrium before illuminating the CR solution to solar light. During the photocatalytic experiment, the light intensity and temperature were between 12450-13174 Lux and 32–43 °C, respectively. The effect of initial solution pH was performed between pH 5 to 9 because below pH 4, CR solution showed the color change due to the tautomerism. The CR solution pH was adjusted using 0.1 M HCl or NaOH. Moreover, the effect of the initial CR concentrations on photocatalytic process was performed at the range of 10 to 50 mg/L. The samples were collected on regular intervals, filtered with 0.22 μm syringe filter to remove the catalyst and analyzed by the UV-visible spectrophotometer (UV 1800 Shimadzu) at 497 nm.
